# Prediction of brain metastasis in patients with epidermal growth factor receptor-positive lung adenocarcinoma based on lung computed tomography-derived radiomics features

**DOI:** 10.1186/s12880-025-02059-4

**Published:** 2025-12-29

**Authors:** Jinhua Zhang, Wei Guo, Lijuan Lin, Xiang Lin, Yang Song, Dairong Cao, Dehua Chen

**Affiliations:** 1 Department of Radiology, The First Affiliated Hospital of Fujian Medical University, 20 Cha-Zhong Road, Fuzhou, Fujian 350005 China; 2 Department of Radiology, National Regional Medical Center, Binhai Campus of the First Affiliated Hospital, Fuzhou, 350212 China; 3 MR Scientific Marketing, Siemens, Healthineers Ltd, Shanghai, 201318 China; 4 Department of Radiology, Fujian Key Laboratory of Precision Medicine for Cancer, The First Affiliated Hospital, Fujian Medical University, Fuzhou, Fujian 350005 China; 5 Key Laboratory of Radiation Biology of Fujian Higher Education Institutions, The First Affiliated Hospital, Fujian Medical University, Fuzhou, Fujian 350005 China

**Keywords:** Brain metastasis, Lung adenocarcinoma, Radiomics, Epidermal growth factor receptor

## Abstract

**Purpose:**

We investigated lung computed tomography (CT) radiomics features feasibility for brain metastasis (BM) prediction in patients with epidermal growth factor receptor-positive lung adenocarcinoma (LA-EGFRp).

**Methods:**

Lung CT images and clinical data of patients were retrospectively analyzed. Patients were classified into BM and non-BM groups, and randomly divided into training and test sets (8:2 ratio). Clinical and CT radiomics features were extracted and trained with various machine-learning classifiers to construct the clinical, radiomics, and hybrid models, respectively. Model performance was assessed using receiver operating characteristic curves.

**Results:**

Among 198 included patients, 72 developed BM. Areas under the curve (AUCs) for predicting BM in the training and test sets were 0.781 and 0.701, 0.989 and 0.865, and 0.957 and 0.929 for the clinical, radiomics, and hybrid models, respectively. The AUCs of the radiomics and hybrid models were significantly higher in the training set (*P* < 0.001) and that of the hybrid model in the test set was higher compared with the clinical model (*P* < 0.05).

**Conclusions:**

Models based on clinical data, lung CT-derived radiomics features, and the two combined predicted BM in LA-EGFRp. Combining radiomics and clinical features significantly improved BM prediction, thereby providing an effective tool for clinical decision-making.

**Supplementary Information:**

The online version contains supplementary material available at 10.1186/s12880-025-02059-4.

## Introduction

Lung cancer is the leading cause of cancer-related deaths worldwide [1]. Among them, the adenocarcinoma form of non-small cell lung cancer (NSCLC) is the most common, accounting for over 50% of all lung cancers [2, 3]. The brain is a common site for lung cancer metastasis [4]. The incidence of brain metastasis (BM) in patients with NSCLC can reach up to 50%, with the highest incidence of BM in cases of epidermal growth factor receptor-positive lung adenocarcinoma (LA-EGFRp) at approximately 45–52% [5, 6]. BM can cause a severe reduction in quality of life and is also the most crucial factor affecting overall survival (median overall survival range: 3.7–19.1 months) [7]. Although many treatment methods for BM of NSCLC are available, their overall efficiency is poor. Compared with targeted interventions after BM has already developed in patients with NSCLC, preventing the development of BM can fundamentally and significantly improve their outcomes. Therefore, timely identification of patients with lung cancer at higher risk of BM for prophylactic treatment is of considerable clinical value.

Previous studies have attempted to predict BM in patients with NSCLC based on clinical data [8–11]. However, these models are based on clinical data (e.g., adjuvant chemotherapy, smoking history) and postoperative pathologic features (e.g., pathology type, peripheral invasion, pathological T and N stages), which are affected by specific trailing indicators and subjective factors, causing discrepant predictive efficiency (area under the curve [AUC] = 0.670–0.844). Nevertheless, the pathology status was obtained using an invasive method or post-treatment, and the treatment strategies were invasive. Therefore, early and non-invasive prediction of BM from lung cancer remains challenging.

The concept of radiomics was proposed by Lambin et al. in 2012 and has become a popular research topic in medical imaging over the past 10 years [12]. Compared with conventional imaging, radiomics can be used for objective and comprehensive investigation of high-throughput features from radiological images (which are invisible to the naked human eye) and provide useful information for tumor diagnosis, prognosis prediction, and molecular typing [13–15]. Researchers have used computed tomography (CT)-derived radiomics features of primary lung cancer lesions to predict BM development and have achieved preliminary results [16–19]. These studies show that the radiomics features of primary lung lesions are closely associated with distant metastasis. However, the histological types of tumors in these studies varied widely, and significant heterogeneity has been reported in the pathologic and imaging features of different lung cancer types. Furthermore, epidermal growth factor receptor mutation status is strongly associated with BM in patients [20, 21]. In conclusion, the feasibility of lung CT-derived radiomics features for predicting BM in patients with LA-EGFRp remains partially elucidated, and relevant reports are currently scarce.

Therefore, we aimed to investigate the feasibility of using lung CT-derived radiomics features to predict the development of BM in patients with LA-EGFRp.

## Materials and methods

### Participants

This study was approved by the Institution Ethics Committee of [BLINDED] in accordance with the Declaration of Helsinki, which waived the requirement for written informed consent owing to the retrospective nature of the study. The data of patients with pathologically confirmed LA-EGFRp between January 2020 and December 2022 at our institution were retrospectively collected. Inclusion criteria were as follows: (1) lesions pathologically confirmed as LA-EGFRp; (2) patients who underwent CT examination of the lungs before treatment. Exclusion criteria: (1) history of other extrapulmonary primary malignancies; (2) concomitant NSCLC with other genetic subtypes; (3) poorly delineated lesion margins with interference from motion artifacts, obstructive pneumonitis, or large amounts of pleural effusion; (4) tumors with a maximum diameter < 1 cm [22]; and (5) other concomitant intracranial enhancing lesions or history of other primary tumors of the central nervous system. All patients were randomly divided into training and test sets at an 8:2 ratio.

### Imaging protocol

Patients underwent a preoperative CT of the lungs, which was performed according to our standard CT scanning protocol using the following CT scanners: Aquilion ONE (Toshiba, Tokyo, Japan; 320-detector row helical CT scanner), Aquilion TSX-101 A (Toshiba, Tokyo, Japan; 64-detector row helical CT scanner), Aquilion TSX-302 A (Toshiba, Tokyo, Japan; 80-detector row helical CT scanner), and IQon Spectral CT (Philips, Amsterdam, Netherlands, dual-energy 64 detector-row helical CT scanner). Scanning parameters: tube voltage, 120 kV; automatic tube current modulation; matrix, 512 × 512; and slice thickness, 0.5/0.625 mm, pitch < 1. A high-resolution algorithm was used for lung window image reconstruction of all acquired raw data. Images were reconstructed into 1.25- or 2-mm transverse axial and coronal data using multiplanar reconstruction.

### Clinical data and BM status identification

Baseline clinical characteristics of patients were collected, including age, sex, tumor site, smoking history, extracranial metastases, adjuvant treatment modalities, and surgical status. In addition, tumors were staged according to the TNM Classification of Malignant Tumours, 8th edition, issued by the Union for International Cancer Control [23].

Brain metastatic lesions were confirmed through pathology and/or neurological imaging, which included magnetic resonance imaging with enhancement during the follow-up phase. Patients underwent surveillance for brain metastases at intervals of 3 to 6 months. Imaging results were evaluated by two radiologists together (with 3 and 5 years of experience in neurological radiology, respectively) and verified by a senior radiologist (with 25 years of experience in neurological radiology) who was unaware of the clinical and lung CT findings. BM was considered positive when typical imaging features of the condition were observed (including single or multiple enhancing lesions in the gray-white matter junction or cerebral watershed area, which is usually solid and nodular, or cystic in patients with LA-EGFRp and small lesions surrounded by large patches of vasogenic edema); and/or when a new lesion was observed on follow-up magnetic resonance imaging; and/or when a lesion suspected to be a metastatic tumor increased in size; and/or when the lesion size decreased after treatment [24, 25]. The follow-up endpoint of this study was February 2023.

### Image preprocessing and tumor segmentation

Before tumor segmentation, the CT transverse-axial images of all patients were uniformly resampled to 1 × 1 × 2 mm with trilinear interpolation to minimize the influence of differences in spatial resolution on the radiomics features of the images.

The volume of interest (VOI) was delineated in a blinded manner by a radiologist with 3 years of experience in diagnostic chest imaging. Lesions were manually delineated layer-by-layer on resampled axial CT images using open-source software (ITK-SNAP version 3.8.0, http://www.itksnap.org) [26]. The VOI includes all lesion components, including cystic, necrotic, and calcified areas, and avoids non-tumor areas, such as large blood vessels, bronchi, bone, and mediastinum.

### Extraction of radiomics features

Quantitative radiomics feature extraction of the CT images in the VOI was performed using FeAture Explore software (FAE, version 0.5.5), which is based on the pyradiomics Python module (version 3.7.6; Python Software Foundation, Wilmington, DE, USA) [27]. For each VOI, feature extraction was performed on the original image and eight wavelet transform images. The radiomics features extracted from the original image included 14 shape-based, 18 first-order statistical, and 75 texture features. In addition, 18 first-order statistical and 75 texture features were extracted from each wavelet transform image. Thus, 851 radiomics features were extracted for each VOI. Details of the radiomics features extracted for this study are listed in Table S1 of the Supplementary Material.

### Robustness evaluation of radiomics features

In this study, the interclass correlation coefficient (ICC) was used to evaluate the robustness of the extracted radiomics features. Thirty patients were randomly selected and re-segmented by the same radiologist after 1 month. Moreover, the radiomics features of two regions of interest were extracted for each patient to calculate the intra-observer ICCs for each feature. Subsequently, another radiologist (with 2 years of experience in diagnostic chest imaging) drew the VOIs of the 30 selected patients, and the inter-observer ICCs for each feature were calculated based on the features extracted from the VOIs delineated by two radiologists. Only radiomics features with simultaneous intra- and inter-observer ICCs > 0.8 were retained for further study.

### Screening of radiomics features

Owing to the unbalanced number of BM and non-BM cases in the training set, data balancing of the training set data was performed through up sampling, resulting in the same number of BM and non-BM cases in the balanced training set. Subsequently, each feature vector was subtracted by the mean value of the vector and divided by its length. In addition, given the high dimensionality of the radiomics feature space, the principal component analysis (PCA) was used to transform high-dimensional into relatively low-dimensional features. Prior to model development, recursive feature elimination was used for feature selection. To avoid model overfitting, the range of the number of features selected was set to be no more than 10% of the sample size of this study, which was 20.

### Model construction

Clinical model construction: Univariate analysis of the clinical characteristics in the training set was conducted, and characteristics with statistically significant differences between the BM and non-BM groups (*P* < 0.05) were screened for clinical model construction. Similarly, upsampling methods were used for data balancing. A five-fold cross-validation of the training set data was used to determine the hyperparameters. Logistic regression was used as a classifier. One-hot encoding of categorical data was performed before analysis.

Radiomics model construction: Six machine learning classifiers, namely logistic regression, random forest, linear discriminant analysis, AdaBoost, decision tree, and naive Bayes, were incorporated for radiomics model construction. A five-fold cross-validation of the training set data was used to automatically determine the hyperparameters resulting in a robust model. The model with the highest AUC in the cross-validation set was selected as the optimal model for constructing the radiomics model. The prediction value for the development of BM in each case was automatically calculated using the final optimal model.

Hybrid model construction: Clinical features with statistically significant differences between groups in the training set were combined with the prediction values of the radiomics model to construct a hybrid model using a method similar to that used to construct the clinical model.

**Fig. 1:**
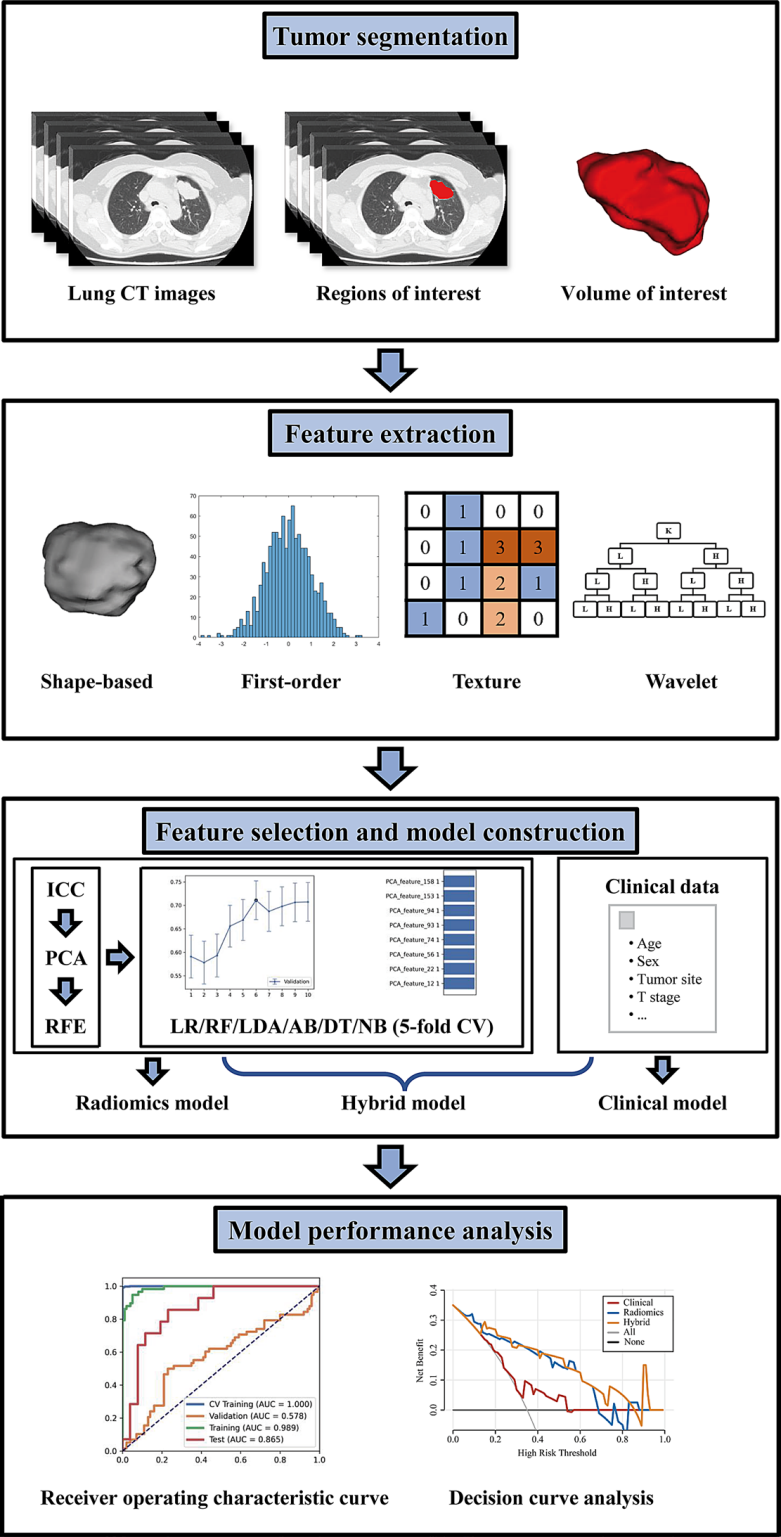
Pipeline of this study. CT, computed tomography; ICC, interclass correlation coefficient; PCA, principal component analysis; RFE, recursive feature elimination; LR, logistic regression; RF, random forest, LDA, linear discriminant analysis, AB, AdaBoost, DT, decision tree; NB, naive Bayes, CV, cross-validation

Feature extraction, data balancing, feature normalization, PCA, recursive feature elimination, and model construction were performed using FeAture Explorer Pro software (FAE, version 0.5.5) based on Python (version 3.7.6). Figure 1 shows a flowchart of the study.

### Statistics

Statistical analysis was performed using R software (version 3.5.3, R Foundation for Statistical Computing, Vienna, Austria). Differences in clinical data between groups were analyzed using the Mann–Whitney U test or Chi-square test. The performance of the models was evaluated using receiver operating characteristic curves. The AUCs were calculated, optimal cutoff values were determined using the maximum Youden index, and the accuracy (ACC), sensitivity (SEN), specificity (SPE), positive predictive, and negative predictive values were calculated. The 95% confidence intervals of the AUCs were estimated using the DeLong test for comparing model performance. Decision curve analysis was also used to evaluate and compare the performance of the models. All *P*-values were two-sided, and differences with *P* < 0.05 were considered statistically significant. Bonferroni correction was used for *post-hoc* comparison of *P-*values.

## Results

### Baseline characteristics

Based on the inclusion and exclusion criteria, 198 patients with LA-EGFRp were included in the study, of whom 72 and 126 were in the BM and non-BM groups, respectively. Brain metastases status was initially confirmed by MRI in 192 patients and by contrast-enhanced CT in six. All enrolled patients subsequently underwent MRI for follow-up evaluations. Forty-three patients had brain metastases at the time of initial tumor staging. A total of 158 patients were included in the training set (BM = 58, non-BM = 100) and 40 in the test set (BM = 14, non-BM = 26). No statistically significant differences were observed in baseline characteristics between the training and test sets (all *P* > 0.05) (Table 1). All treatments listed in the Table 1 were administered as initial therapy.

**Table 1:** Baseline characteristics of patients with and without brain metastasis (BM) in the training and test dataset

Characteristic	Training set (*n* = 158)			Test set (*n* = 40)			*P*_c_-value
	BM (*n* = 58)	Non-BM (*n* = 100)	*P*_a_-value	BM (*n* = 14)	Non-BM (*n* = 26)	*P*_b_-value	
Age (years)	61.74 ± 9.89	62.11 ± 11.25	0.660	59.86 ± 11.42	59.54 ± 12.27	0.887	0.406
Sex, No. (%)			0.520			0.666	0.392
Male	28 (48.28)	43 (43.00)		8 (57.14)	13 (50.00)		
Female	30 (51.72)	57 (57.00)		6 (42.86)	13 (50.00)		
Tumor site, No. (%)			0.399			0.083	0.727
Left lung	23 (39.66)	33 (33.00)		7 (50.00)	6 (23.08)		
Right lung	35 (60.34)	67 (67.00)		7 (50.00)	20 (76.92)		
T stage, No. (%)			**0.047**			0.490	0.545
T1	16 (27.59)	38 (38.00)		3 (21.43)	12 (46.15)		
T2	11 (18.97)	31 (31.00)		4 (28.57)	5 (19.23)		
T3	15 (25.86)	16 (16.00)		2 (14.29)	3 (11.54)		
T4	16 (27.59)	15 (15.00)		5 (35.71)	6 (23.08)		
Smoking history, No. (%)			0.607			0.320	0.972
None	45 (77.59)	81 (81.00)		10 (71.43)	22 (84.62)		
Smoker	13 (22.41)	19 (19.00)		4 (28.57)	4 (15.38)		
Extracranial metastasis, No. (%)			0.201			0.136	0.965
No	14 (24.14)	38 (38.00)		2 (14.29)	11 (42.31)		
Yes	40 (68.97)	56 (56.00)		10 (71.43)	14 (53.85)		
Unknown	4 (6.90)	6 (6.00)		2 (14.29)	1 (3.85)		
Adjuvant therapy, No. (%)			**< 0.001**			0.103	0.738
None	19 (32.76)	69 (69.00)		4 (28.57)	19 (73.08)		
Radiotherapy	1 (1.72)	2 (2.00)		1 (7.14)	1 (3.85)		
Chemotherapy	7 (12.07)	4 (4.00)		2 (14.29)	1 (3.85)		
Targeted therapy	16 (27.59)	15 (15.00)		5 (35.71)	3 (11.54)		
Combination therapy	15 (25.86)	10 (10.00)		2 (14.29)	2 (7.69)		
N stage, No. (%)							
N0	10 (17.24)	38 (38.00)	**0.004**	2 (14.29)	7 (26.92)	0.506	0.545
N1	4 (6.90)	10 (10.00)		0 (0.00)	2 (7.69)		
N2	18(31.03)	25 (25.00)		4 (28.57)	6 (23.08)		
N3	18 (31.03)	10 (10.00)		6 (42.86)	5 (19.23)		
Nx	8 (13.79)	17 (17.00)		2 (14.29)	6 (23.08)		
Tumor resection No. (%)	5 (8.62)	30 (30.00)	**0.002**	1 (7.14)	6 (23.08)	0.206	0.520

Note: *P*_a_ represents the statistically significant level of the difference between the two groups in the training set, *P*_b_ represents the statistically significant level of the difference between the two groups in the test set, and *P*_c_ represents the statistically significant level of the feature distribution difference between the training group and the test group. The bold type indicated a statistical significance difference between groups (*P* < 0.05)

### Clinical model construction and predictive efficiency evaluation

**Fig. 2:**
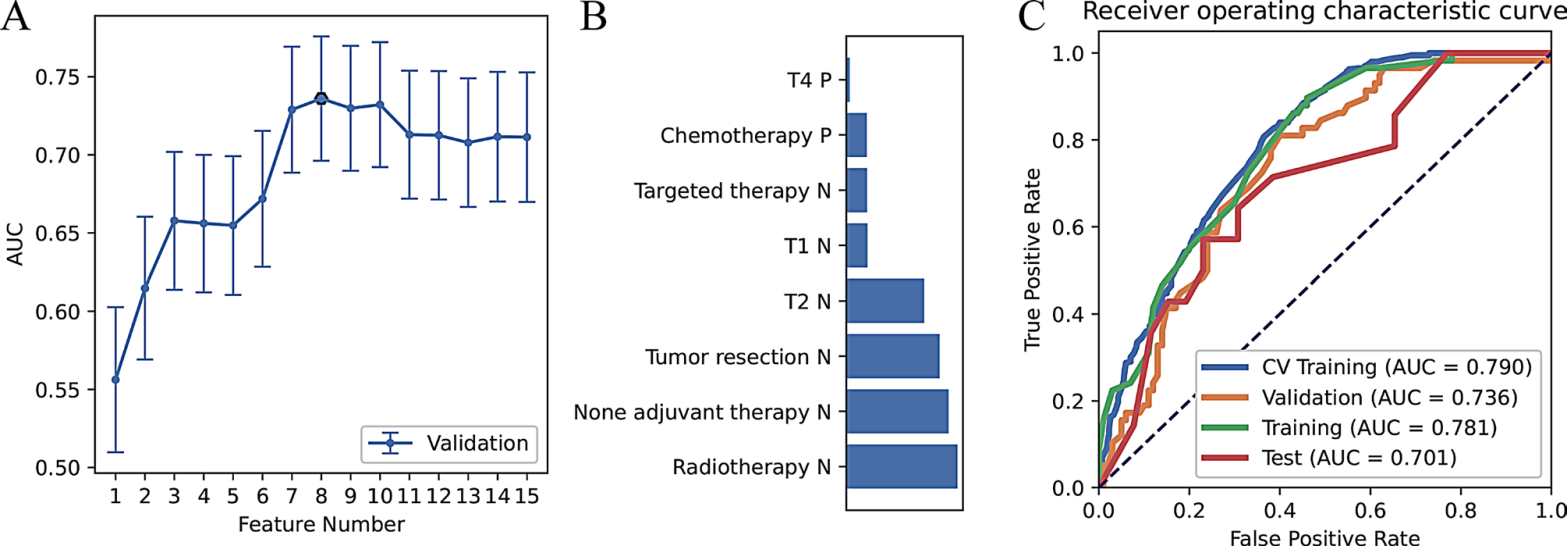
Clinical model performance. (**A**) The area under the curve (AUC) value for cross-validation of various feature numbers; (**B**) feature weight; and (**C**) receiver operating characteristic (ROC) curves of different datasets. CV, cross-validation

In the training set, the differences in T stage, adjuvant treatment modalities, and surgical status between the BM and non-BM groups were statistically significant (all *P* < 0.05) (Table 1). Based on the cross-validation set results, the T2 stage, T4 stage, radiotherapy, targeted therapy, combination therapy, and surgical status were selected as model features (Fig. 2A and B). The model had the following values: AUC = 0.781, ACC = 0.690, SEN = 0.724, and SPE = 0.670 in the training set and AUC = 0.701, ACC = 0.675, SEN = 0.643, and SPE = 0.692 in the test set (Table 2; Fig. 2C).

**Table 2:** Predictive performance of the clinical, radiomics, and hybrid models

Model	Cohort	AUC	95%CI	Cut-off	ACC	SEN	SPE	PPV	NPV
Clinical model	Training set	0.781	0.710–0.851	0.482	0.690	0.724	0.67	0.56	0.807
	Test set	0.701	0.531–0.870	0.482	0.675	0.643	0.692	0.529	0.783
Radiomics model	Training set	0.989	0.978-1.000	0.500	0.943	0.931	0.950	0.915	0.960
	Test set	0.865	0.751–0.980	0.500	0.775	0.500	0.923	0.778	0.774
Hybrid model	Training set	0.957	0.930–0.984	0.518	0.899	0.931	0.880	0.818	0.957
	Test set	0.929	0.854-1.000	0.518	0.800	0.571	0.923	0.800	0.800

Note: AUC, area under the curve; CI, confidence interval; ACC, accuracy; SEN, sensitivity; SPE, specificity; PPV, positive predictive value; NPV negative predictive value

### Radiomics model construction and predictive efficiency evaluation

**Fig. 3:**
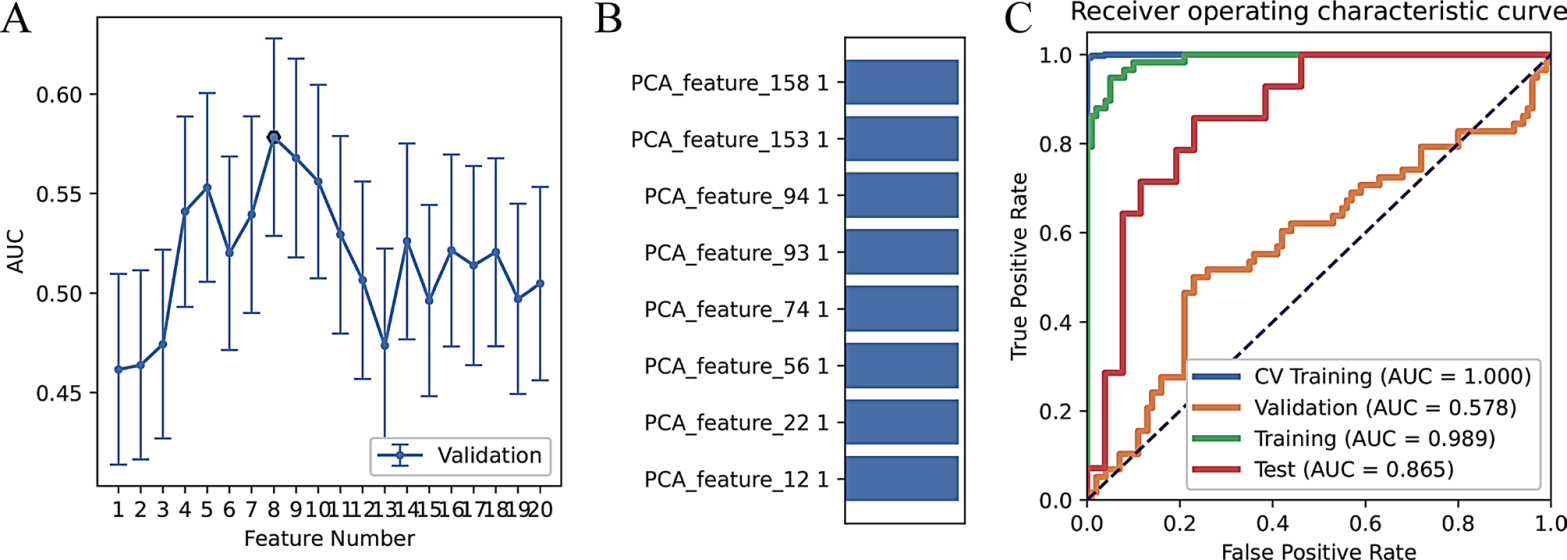
Radiomics model performance. (**A**) The area under the curve (AUC) value for cross-validation of various feature numbers; (**B**) feature weight; and (**C**) receiver operating characteristic (ROC) curves of different datasets. PCA, principal component analysis; CV, cross-validation

Among the six classifiers, the model based on the AdaBoost classifier had the highest efficiency in the cross-validation set, suggesting the best model fit and robustness (Table S2). The AdaBoost classifier-based model incorporated eight features after PCA dimensionality reduction (Fig. 3A and B). The model had the following values: AUC = 0.989, ACC = 0.943, SEN = 0.931, and SPE = 0.950 in the training set and AUC = 0.865, ACC = 0.775, SEN = 0.500, and SPE = 0.923 in the test set (Fig. 3C; Table 2).

### Hybrid model construction and predictive efficiency evaluation

**Fig. 4:**
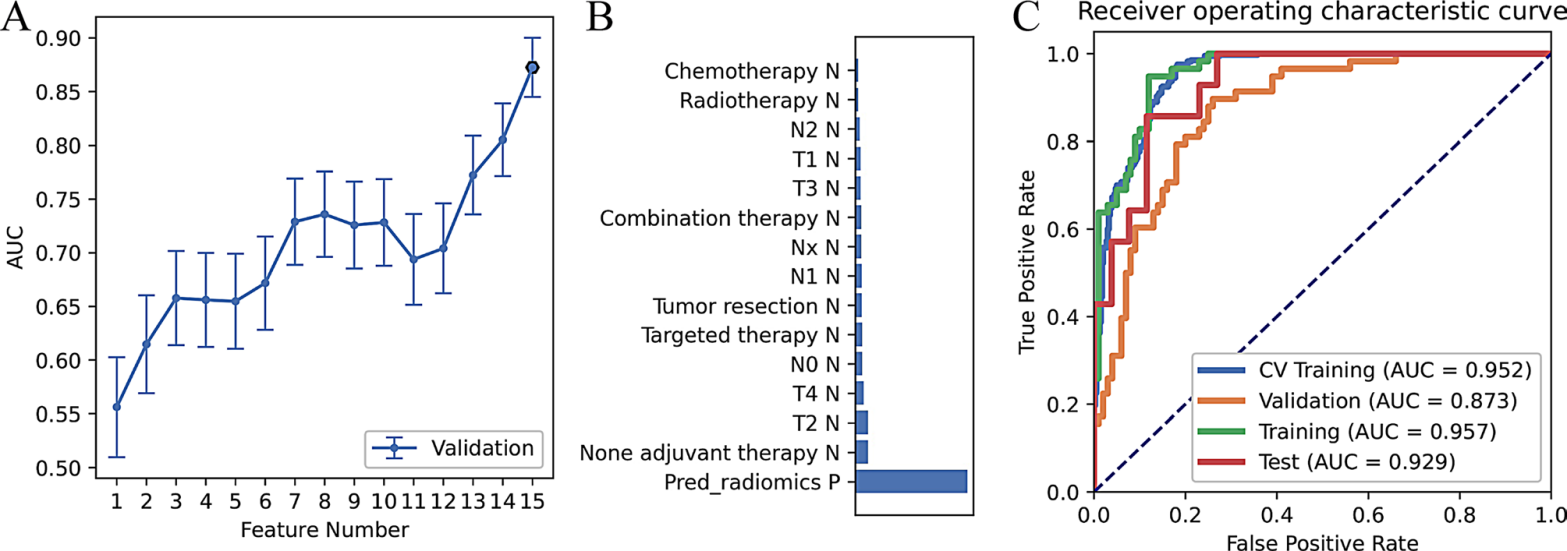
Hybrid model performance. (**A**) The area under the curve (AUC) value for cross-validation of various feature numbers; (**B**) feature weight; and (**C**) receiver operating characteristic (ROC) curves of different datasets. CV, cross-validation

The model with 8 incorporated features had the highest AUC in the cross-validation set (Fig. 4A and B) (AUC = 0.736). The model had the following values: AUC = 0.957, ACC = 0.899, SEN = 0.931, and SPE = 0.88 in the training set and AUC = 0.929, ACC = 0.800, SEN = 0.571, and SPE = 0.923 in the test set (Fig. 4C; Table 2).

### Comparison of predictive efficiency among three models

In the training set, both the radiomics and hybrid models had significantly better predictive power than did the clinical model (*P* < 0.001). However, only the predictive efficiency of the hybrid model was significantly better than that of the clinical model in the test set (*P* = 0.026) (Table 3).

**Table 3:** Comparison results of the predictive performance among models according to DeLong test results

Cohort	Model	Z-value	*P*-value (Adjusted)
Training set	Radiomics model vs. Clinical model	5.653	**< 0.001**
	Radiomics model vs. Hybrid model	2.252	0.073
	Hybrid model vs. Clinical model	5.946	**< 0.001**
Test set	Radiomics model vs. Clinical model	1.487	0.411
	Radiomics model vs. Hybrid model	1.767	0.231
	Hybrid model vs. Clinical model	2.622	**0.026**

Note: The bold type indicated a statistical significance difference between groups (*P* < 0.05). The Bonferroni correction was used for *post-hoc* multiple testing *P-*values

**Fig. 5:**
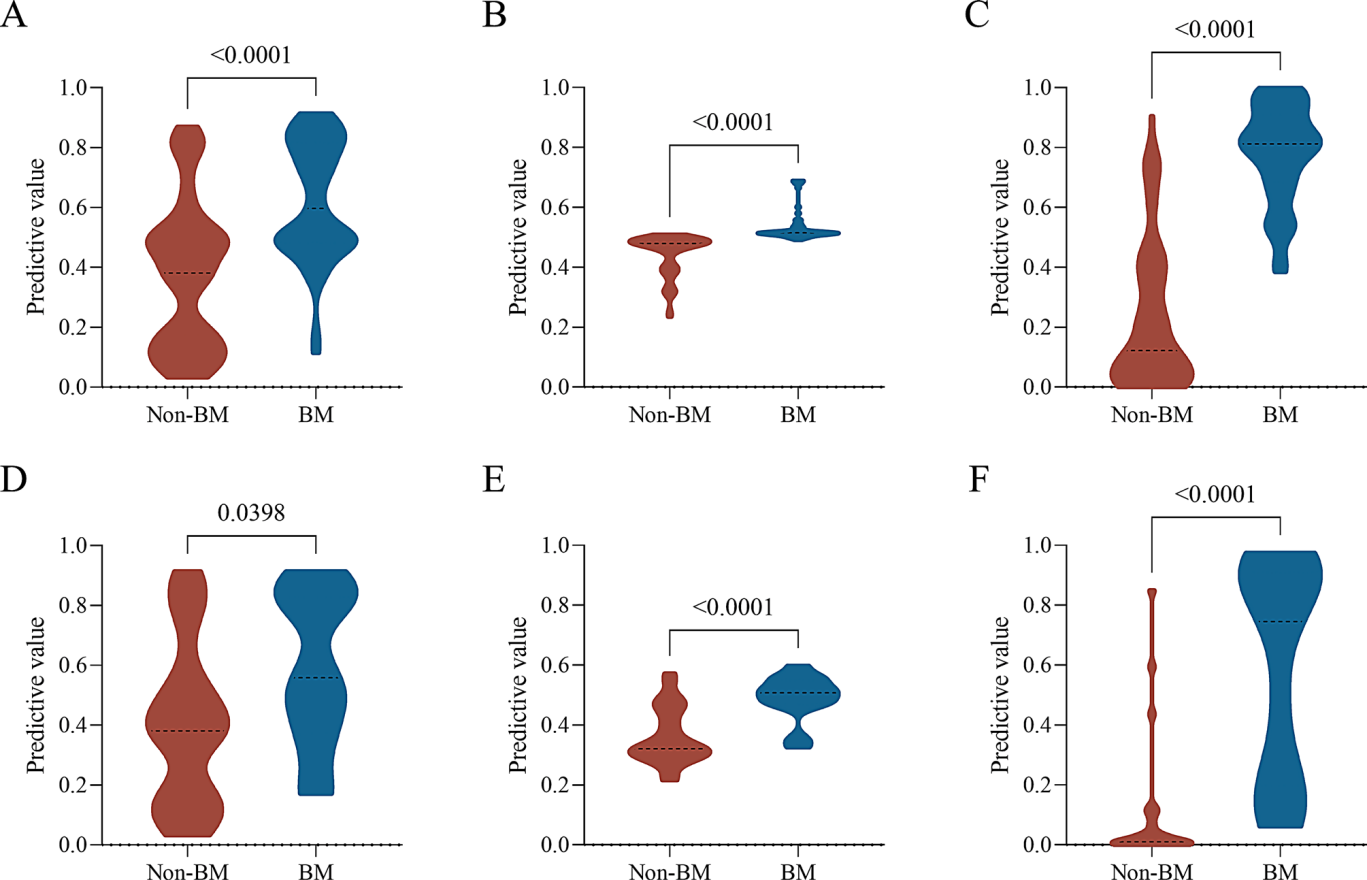
Distribution of predictive values of the three models between brain metastasis (BM) and non-BM groups. Distribution of predictive values of clinical (**A**), radiomics (**B**), and hybrid model (**C**) in the training set; distribution of predictive values of clinical (**D**), radiomics (**E**), and hybrid model (**F**) in the test set

In the training set, the differences in predictive values among all three models were statistically significant between the BM and non-BM groups (all *P* < 0.001). In the test set, only the differences in predictive values of the radiomics and hybrid models were statistically significant between the BM and non-BM groups (all *P* < 0.001) (Fig. 5).

**Fig. 6:**
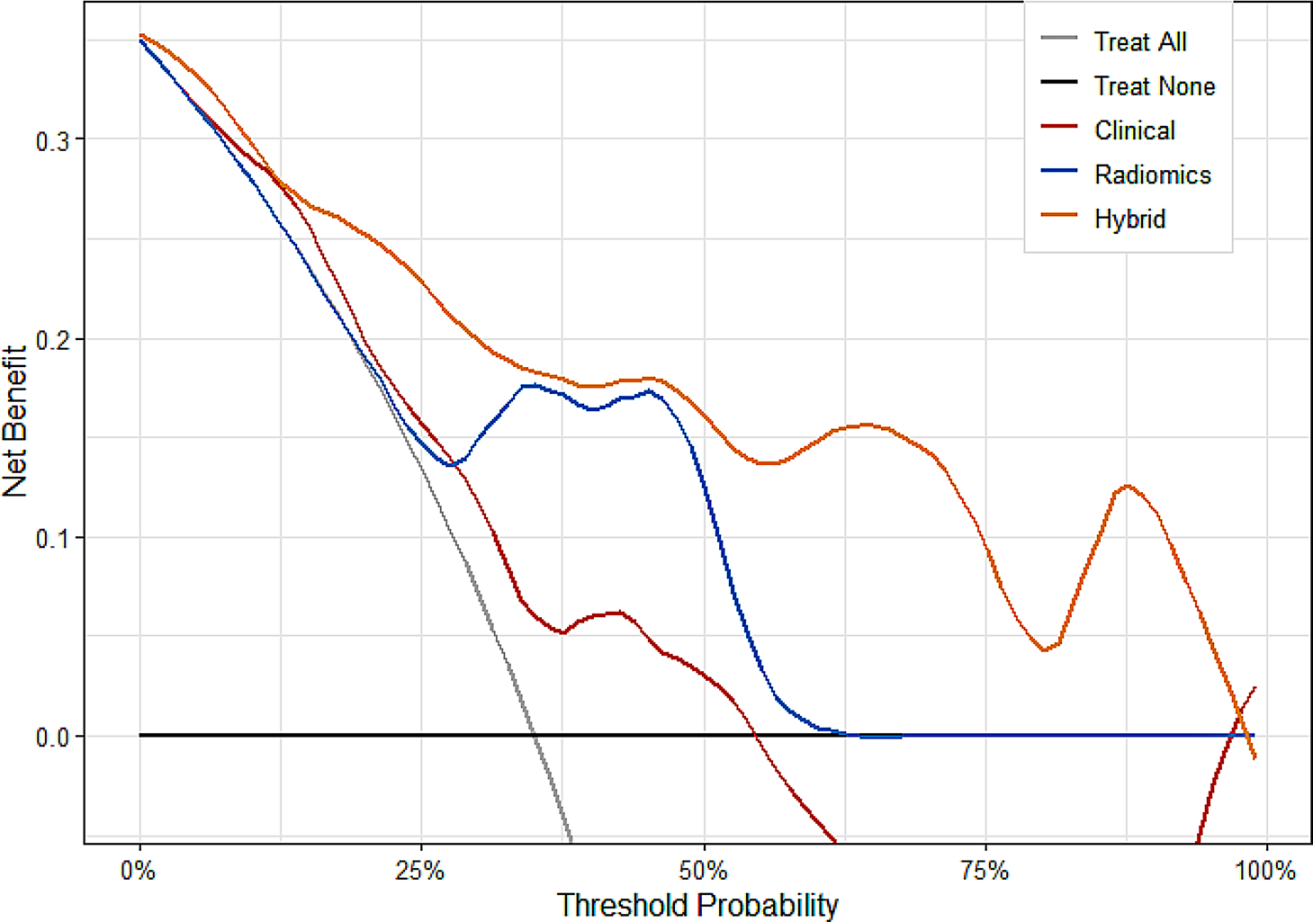
Decision curve analysis of the tree predictive models in the testing cohorts. The x-axis indicates the threshold probability, while the y-axis indicates the net benefit. The gray line indicates the hypothesis that all the patients had brain metastasis (BM), whereas the black line indicates the hypothesis that all the patients had non-BM

Furthermore, in the test set, decision curve analysis revealed that the hybrid model resulted in a greater net benefit than the other two models in predicting BM in patients with LA-EGFRp (Fig. 6).

## Discussion

In this study, the efficiency of clinical data and CT-derived radiomics features in predicting BM status in patients with LA-EGFRp was retrospectively analyzed. The results showed that predictive models based on clinical data and/or CT radiomics features could be used to predict BM status in patients with LA-EGFRp in a non-invasive manner (test set, AUC = 0.701–0.929). Among them, the predictive efficiency of the CT-derived radiomics features-based model was superior to that of the clinical features-based model (test set, AUC = 0.865 vs. 0.701). Combining clinical data and CT radiomics features further improved the predictive efficiency of the model (test set, AUC = 0.929 vs. 0.701).

In previous studies, the clinical data of patients with NSCLC were used to construct preliminary models for predicting BM status in patients with lung cancer [8–11]. In the clinical model of our study, the T stage was a clinical feature predictive of BM, consistent with the results of previous studies [20, 28, 29]. A potential reason is that T4-stage tumors are more locally invasive and have a higher risk of metastasis than do those with T1–3 stages because of a greater extent of peritumoral area involvement. In line with a previous study, our results suggest that adjuvant treatment modalities are closely associated with BM [8]. Among them, targeted and combination therapies are positive risk factors for BM. A prior study yielded similar results and suggested that the reason for the increased incidence of BM was prolonged patient survival with more effective systemic treatment of the primary tumor [21]. Because of the high prevalence of BM in patients with lung cancer, the possibility of developing BM is higher over a longer follow-up period. Notably, surgical status and adjuvant radiotherapy were negative risk factors in our study. Another study also revealed that complete surgical resection could prolong survival and decrease the incidence of BM during follow-up [30]. Surgical resection minimizes the burden of the primary tumor and enables superior control and remission of disease progression. Similarly, adjuvant radiotherapy can be used to further remove residual tumor tissue and invasive tumor cells in and around the operative area, reducing the risk of distant tumor metastasis.

Recent research interest in using radiomics to predict distant metastasis in lung cancer has gradually increased. Previous studies have demonstrated high feasibility for predicting distant metastasis (e.g., lymph node metastasis and BM) of lung cancer based on CT-derived radiomics features of the primary lung lesion, which is consistent with the results of the present study [17, 19, 31, 32]. The CT-derived radiomics features of the primary lung lesion may reflect the internal heterogeneity and microstructure of the lesion, with these internal features being closely associated with the aggressiveness of the tumor [33]. High tumor heterogeneity allows tumors to exhibit varied treatment responses to clinical outcomes, potentially leading to a higher risk of treatment resistance and development of BM. Tumor invasion of microstructures and adjacent tissues is closely associated with the probability of tumor metastasis. Although high-resolution CT imaging can depict the structural features of the tumor, the microstructural changes in and around the tumor cannot be accurately determined with the naked eye alone. Radiomics features can be used for quantitative characterization of such heterogeneity-related and microstructural features of tumors [34]. Therefore, including radiomics techniques can efficiently predict BM in patients with lung cancer.

Our results showed that the predictive efficiency of the lung CT-derived radiomics model was higher than that of the clinical model. Moreover, combining the clinical data and CT-derived radiomics features could further improve the predictive efficiency of BM and provide more benefits than the other two did for clinical practice. In addition, these results were consistent with those of previous studies using CT-derived radiomics features of primary lung lesions to predict BM and metastasis to other sites [17, 19, 31]. Radiomics features revealed the heterogeneity and microstructure of the tumor with numerous quantification features, which were more comprehensive and robust than clinical features. Nevertheless, the clinical features were obtained using invasive procedures such as biopsy or resection, or post-surgical treatment, which may be potentially influenced by other factors such as surgical operator or individual inherent differences. This may indicate the superiority of the radiomics model over the clinical model. However, we cannot deny that clinical factors provide some valuable information, and the combination of these useful factors could be used to estimate the risk of BM in a more comprehensive way than using them solely. Thus, the hybrid model allows their complement, yielding more metastasis-related tumor data, and improving the predictive ability of the combined model.

This study had some limitations. First, this was a single-center retrospective study, which may include some selection bias, and the sample size was relatively small. A prospective, multicenter study with a larger sample size should be conducted for further validation of the model in the future. However, our results provide a valuable direction for subsequent research. The broader underlying biological meaning of CT radiomics features should be the focus of prospective studies. Secondly, molecular imaging has played a crucial role in the evaluation of BM patients over the past few years [35]. Future research may incorporate molecular imaging to explore more potent predictive models. Thirdly, the diagnosis of brain metastases by CT in a subset of patients, rather than MRI, may have limited the detection of smaller lesions, as CT is less sensitive than MRI for this purpose. Finally, variability in follow-up duration may have influenced the observed incidence of brain metastases, as shorter surveillance periods might not fully capture late-onset events. Future studies with uniform MRI-based assessment and standardized follow-up are needed to address these limitations.

## Conclusions

The model based on the lung CT-derived radiomics features outperformed the clinical data-based model for predicting BM in patients with LA-EGFRp. Combining radiomics and clinical features significantly improved BM prediction, and added complementary benefit for clinical decision-making.

## Supplementary Information

Below is the link to the electronic supplementary material.

Supplementary Material 1

## Data Availability

The data that support the findings of this study are available from the corresponding author upon reasonable request.

## Abbreviations

ACC: Accuracy
AUC: Area under the curve
BM: Brain metastasis
CT: Computed tomography
ICC: Interclass correlation coefficient
LA-EGFRp: Epidermal growth factor receptor-positive lung adenocarcinoma
NSCLC: Non-small cell lung cancer
PCA: Principal component analysis
SEN: Sensitivity
SPE: Specificity
VOI: Volume of interest
