# Supplementary material for: Prediction of brain metastasis in patients with epidermal growth factor receptor-positive lung adenocarcinoma based on lung computed tomography-derived radiomics features
Source: BMC Med Imaging. 2025 Dec 29;25:511. doi: 10.1186/s12880-025-02059-4 (PMC12751951; doi:10.1186/s12880-025-02059-4)
Supplement: Supplementary file 1 — Supplementary Material 1 [file 12880_2025_2059_MOESM1_ESM.docx]

**Table S1:** Radiomics features extracted in this study

| **Feature category** | **Feature name** |
| --- | --- |
| **First-order statistics features (n = 18)** | 10 Percentile, 90 Percentile, Energy, Entropy, Interquartile Range, Kurtosis, Maximum, Mean, Mean Absolute Deviation, Median, Minimum, Range, Robust Mean Absolute Deviation, Root Mean Squared, Skewness, Total Energy, Uniformity, Variance |
| **Shape-based features (n = 14)** | Elongation, Flatness, Least Axis Length, Major Axis Length, Maximum 2D Diameter Column, Maximum 2D Diameter Row, Maximum 2D Diameter Slice, Maximum 3D Diameter, Mesh Volume, Minor Axis Length, Sphericity, Surface Area, Surface Volume Ratio, Voxel Volume |
| **Gray-level co-occurrence matrices (GLCM) features (n = 24)** | Autocorrelation, Cluster Prominence, Cluster Shade, Cluster Tendency, Contrast, Correlation, Difference Average, Difference Entropy, Difference Variance, Id, Idm, Idmn, Idn, Imc1, Imc2, Inverse Variance, Joint Average, Joint Energy, Joint Entropy, MCC, Maximum Probability, Sum Average, Sum Entropy, Sum Squares |
| **Gray-level run-length matrices (GLRLM) features (n = 16)** | Gray Level Non-Uniformity, Gray Level Non-Uniformity Normalized, Gray Level Variance, High Gray Level Run Emphasis, Long Run Emphasis, Long Run High Gray Level Emphasis, Long Run Low Gray Level Emphasis, Low Gray Level Run Emphasis, Run Entropy, Run Length Non-Uniformity, Run Length Non-Uniformity Normalized, Run Percentage, Run Variance, Short Run Emphasis, Short Run High Gray Level Emphasis, Short Run Low Gray Level Emphasis |
| **Gray-level size zone matrices (GLSZM) features (n = 16)** | Gray Level Non-Uniformity, Gray Level Non-Uniformity Normalized, Gray Level Variance, High Gray Level Zone Emphasis, Large Area Emphasis, Large Area High Gray Level Emphasis, Large Area Low Gray Level Emphasis, Low Gray Level Zone Emphasis, Size Zone Non-Uniformity, Size Zone Non-Uniformity Normalized, Small Area Emphasis, Small Area High Gray Level Emphasis, Small Area Low Gray Level Emphasis, Zone Entropy, Zone Percentage, Zone Variance |
| **Gray-level dependence matrices (GLDM) features (n = 14)** | Dependence Entropy, Dependence Non-Uniformity, Dependence Non-Uniformity Normalized, Dependence Variance, Gray Level Non-Uniformity, Gray Level Variance, High Gray Level Emphasis, Large Dependence Emphasis, Large Dependence High Gray Level Emphasis, Large Dependence Low Gray Level Emphasis, Low Gray Level Emphasis, Small Dependence Emphasis, Small Dependence High Gray Level Emphasis, Small Dependence Low Gray Level Emphasis |
| **Neighborhood gray-tone difference matrices (NGTDM) features (n = 5)** | Busyness, Coarseness, Complexity, Contrast, Strength |

Note: 2D, two-dimension; Id, inverse difference; Idm, inverse difference moment; Idmn , inverse difference moment normalized; Idn, inverse difference normalized; Imc, informal measure of correlation; MCC, maximal correlation coefficient.

**Table S2:** ROC curve results of six machine learning classifiers in the training and cross-validation sets.

| Patient Set | AUC | | | | | |
| --- | --- | --- | --- | --- | --- | --- |
|  | LDA | RF | LR | AB | DT | NB |
| Training | 0.779 | 1.000 | 0.627 | 0.989 | 1.000 | 0.811 |
| CV training | 0.806 | 1.000 | 0.624 | 1.000 | 1.000 | 0.834 |
| CV validation | 0.539 | 0.575 | 0.542 | **0.578** | 0.528 | 0.527 |

Note：ROC, receiver operating characteristic; CV, cross-validation; AUC, area under the curve; LDA, linear discriminant analysis; RF, random forests; LR, logistic regression; AB, AdaBoost; DT, decision tree; NB, naive Bayes. The bold type indicated the highest AUC in the

CV validation set.
